# Supplementary material for: Single-molecule tracking of Nodal and Lefty in live zebrafish embryos supports hindered diffusion model
Source: Nat Commun. 2022 Oct 15;13:6101. doi: 10.1038/s41467-022-33704-z (PMC9569377; doi:10.1038/s41467-022-33704-z)
Supplement: Supplementary file 1 — Supplementary Information [file 41467_2022_33704_MOESM1_ESM.pdf]

Supplementary Information

Single-molecule tracking of Nodal and Lefty in live zebrafish embryos supports hindered diffusion model

Timo Kuhn[1], Amit N. Landge[2,*], David Mörsdorf[3,4,*], Jonas Coßmann[1], Johanna Gerstenecker[1], Daniel Čapek[2], Patrick Müller[#,2,3] and J. Christof M. Gebhardt[#,1]

[1]Institute of Biophysics, Ulm University, Albert-Einstein-Allee 11, 89081 Ulm, Germany

[2]University of Konstanz, Universitätsstraße 10, 78464 Konstanz, Germany

[3]Friedrich Miescher Laboratory of the Max Planck Society, Max-Planck-Ring 9, 72076 Tübingen, Germany

[4]University of Vienna, Department of Neurosciences and Developmental Biology, Djerassiplatz 1, 1030 Vienna, Austria

[*]These authors contributed equally

[#]To whom correspondence should be addressed:
patrick.mueller@uni-konstanz.de
christof.gebhardt@uni-ulm.de

Contents

Supplementary Figures 2

Supplementary Tables 12

Supplementary Figures

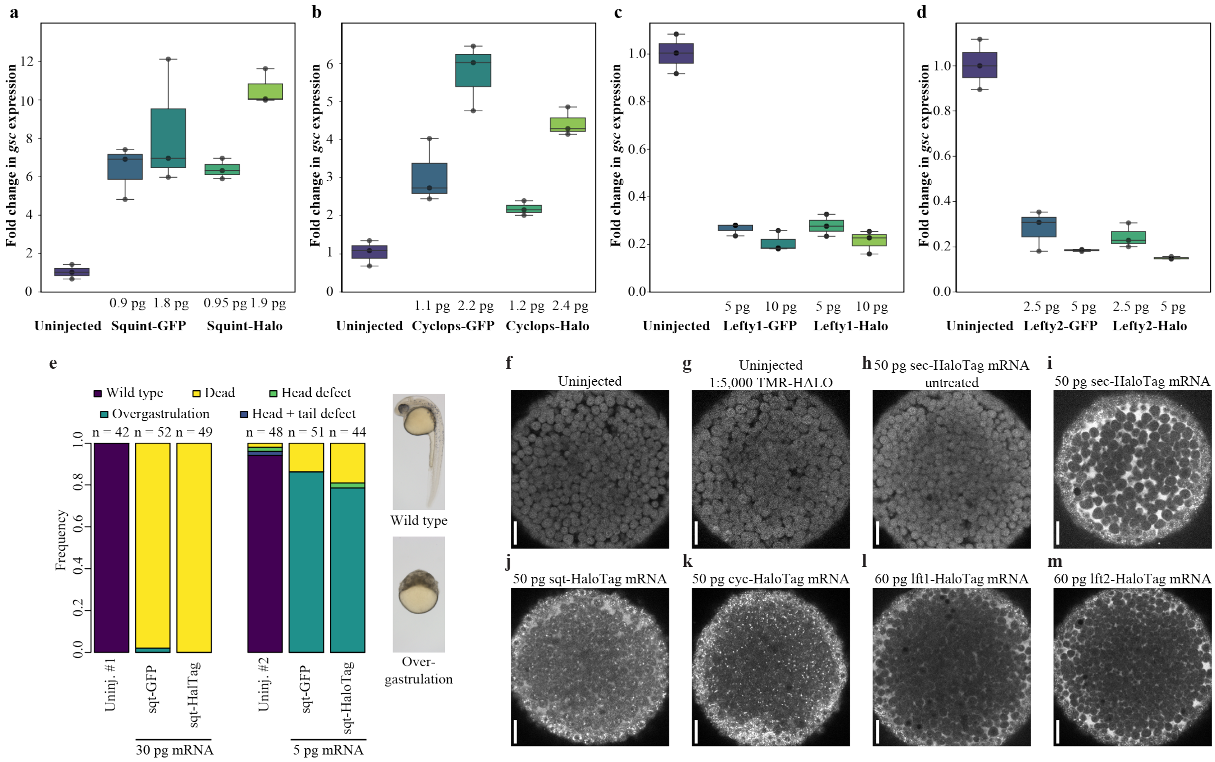

Supplementary Figure 1. Activity and localization of HaloTag fusions. a-d) One-cell stage zebrafish embryos were injected with the indicated equimolar amounts of mRNAs encoding the fusion constructs and collected when their uninjected siblings reached 50% epiboly. Fold change in expression of the Nodal target gene goosecoid (*gsc*) compared to the uninjected embryos is shown for embryos injected with fusion constructs of Nodals (a,b) and Leftys (c,d). The previously characterized GFP-fusion constructs[25,37,39] were used as positive controls. The zebrafish elongation factor *eF1α* was used as a normalization control. Each biological replicate is plotted as a black dot. The center line of the boxplot marks the median, the bounds of the box show interquartile ranges, and the whiskers extend to the minimum and the maximum of the data. n = 3 biological replicates per treatment with ten embryos per biological replicate. **e)** The phenotypes upon injection of squint-GFP or squint-HaloTag mRNA are comparable. High (30 pg mRNA, left) and low (5 pg mRNA, right) expression levels were assessed. **f-m)** Representative optical slices (animal views) of embryos that were uninjected (f, 14 embryos from 3 independent experiments), uninjected but treated with TMR-HALO ligand (g, 3 embryos), injected with secreted-HaloTag-encoding mRNA but not treated with TMR-HALO ligand (h, 2 embryos), and embryos expressing Secreted-HaloTag (i, 4 embryos), Squint-HaloTag (j, 6 embryos), Cyclops-HaloTag (k, 7 embryos), Lefty1-HaloTag (l, 5 embryos), or Lefty2-HaloTag (m, 6 embryos) which were labeled with TMR-HALO ligand. Background fluorescence is similar in unlabeled Secreted-HaloTag-expressing embryos and TMR-HaloTag-treated uninjected embryos, but note that the fluorescence intensities between panels are not comparable due to different detector gain settings. Scale bar = 50 µm. Source data are provided as a Source Data file for Fig. S1a-d.

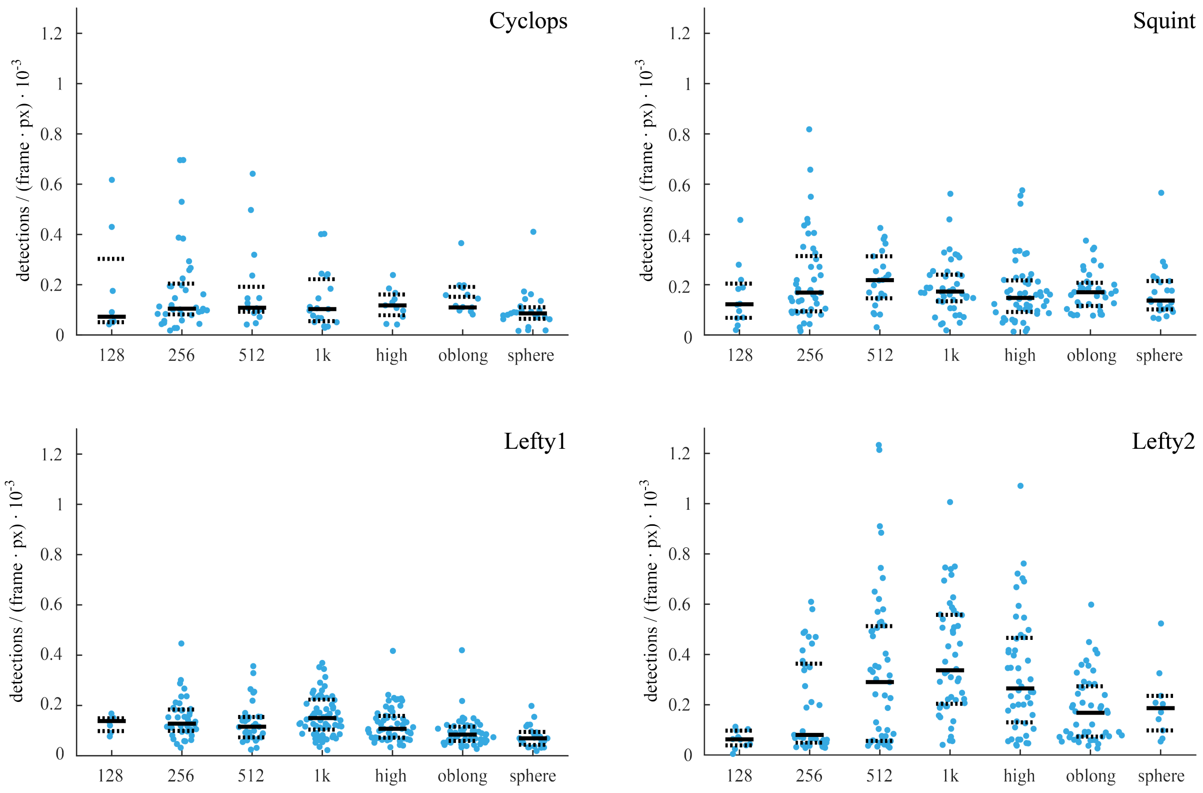

Supplementary Figure 2. Average number of extracellular single molecule detections per frame in each movie. Solid black lines indicate the median values, dashed black lines the 0.25 and 0.75 quantiles. For full experimental statistics see Supplementary Table 7. Source data are provided as a Source Data file.

a

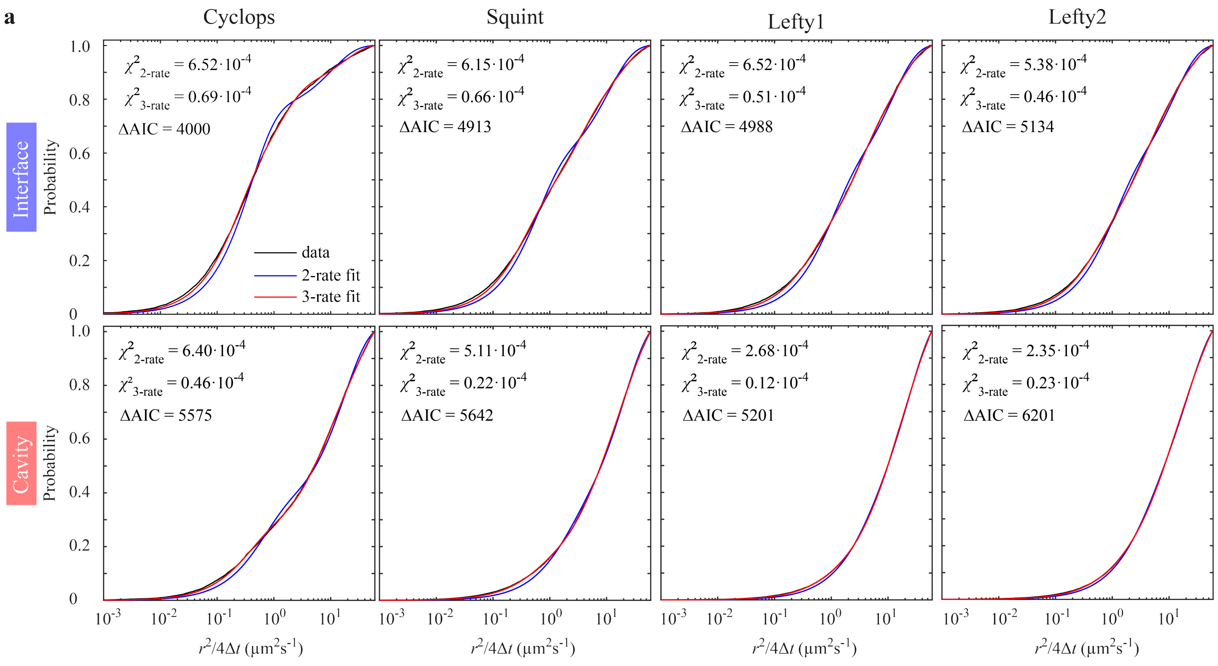

Supplementary Figure 3. **Fitting of jump distance distributions with a 2-rate and 3-rate diffusion model in interface (top) and cavity (bottom) regions for Cyclops, Squint, Lefty1 and Lefty2.** Based on the reduced χ^2 and the Akaike Information Criterion (AIC), a three-component diffusion model best described the data.

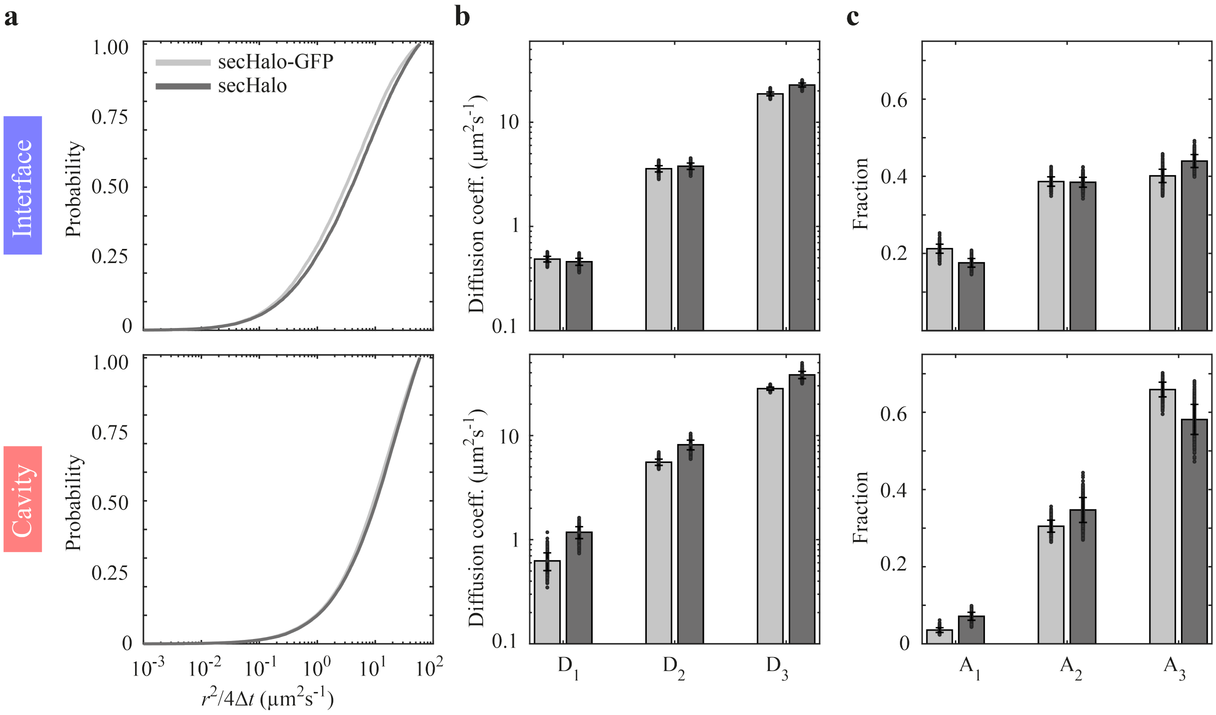

Supplementary Figure 4. Mobility of sec-Halo-GFP and sec-Halo in cell-cell interfaces and extracellular cavities. a) Cumulative distributions of jump distances in interfaces and cavities for sec-Halo-GFP and sec-Halo. **b)** Diffusion coefficients and **c)** fractions of the three-component diffusion model (Supplementary Table 1 and 2). Data are presented as mean values ± s.d. of 500 resamplings with randomly selected 50% of the data. For full experimental statistics see Supplementary Table 5. Source data are provided as a Source Data file.

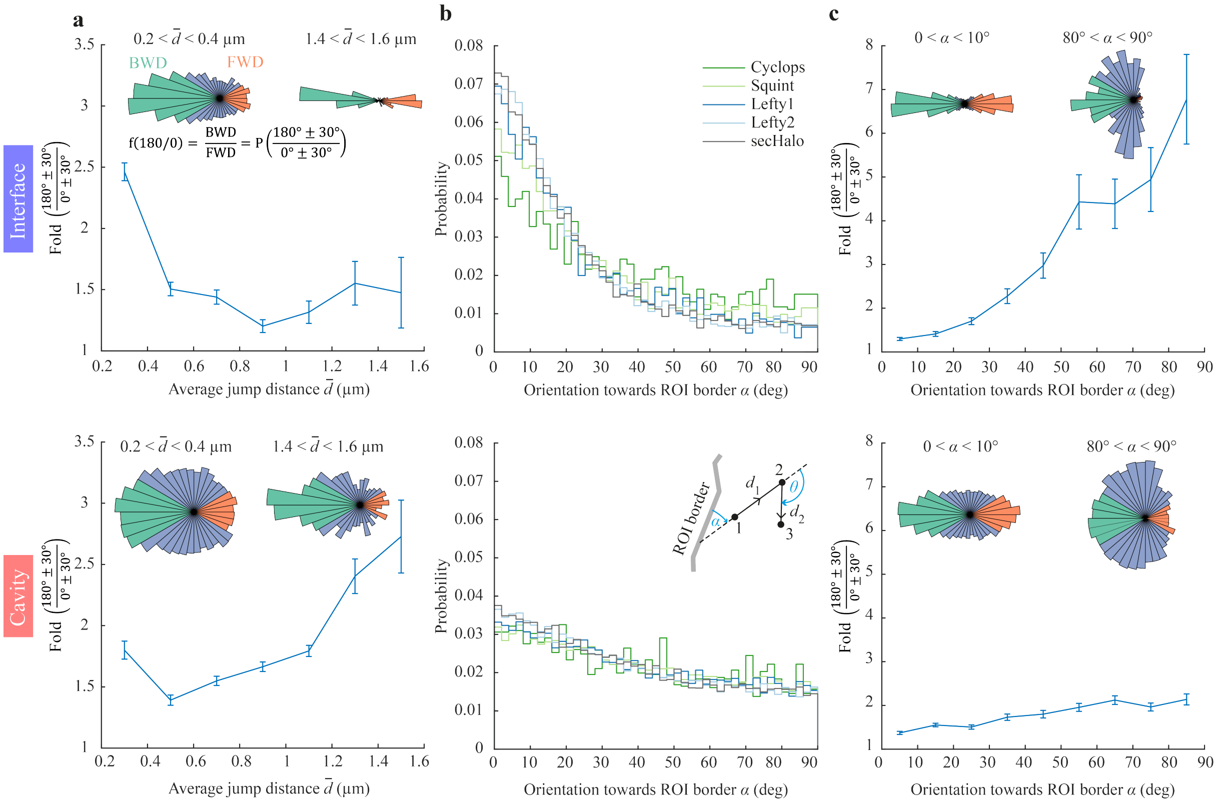

Supplementary Figure 5. Track orientation and jump angles reveal restricted diffusion in interfaces and cavities. a) Fold anisotropy $f_{180/0}$ as a function of the mean jump distance $\overline{d} = \frac{d_1+d_2}{2}$ of the two involved track segments. In interfaces, the anisotropy was higher for small mean jump distances, possibly indicating partial trapping or immobilization. In contrast, the anisotropy increased with larger average jump distances in cavities, reflecting a forced change in direction for higher average jump distances in these regions. Insets: distribution of jump angles for the lowest and highest analyzed mean jump distances. Probabilities for backward and forward motion shown in green and red, respectively. Data of all morphogens and sec-Halo pooled. **b)** Distribution of the orientation of jumps with respect to the nearest ROI border in interfaces and cavities for the indicated morphogen and sec-Halo. Inset: illustration showing the definition of the angle α between track segments and the ROI border, and the jump angle θ towards the subsequent track segment. **c)** Fold anisotropy $f_{180/0}$ as a function of the orientation towards the region border. In interfaces, the anisotropy increases strongly for larger angles due to the reduced dimensionality. For jumps parallel to interface regions, the subsequent jumps were directed comparably in forward or backward direction, whereas jumps at an angle to the ROI border were predominantly followed by a jump in backward direction. This effect is much less pronounced in cavities. Insets: distribution of jump angles of tracks oriented parallel and perpendicular to ROI border. Probabilities for backward and forward motion shown in green and red, respectively. Data of all morphogens and sec-Halo pooled. Center values in a,c) are calculated from the formula using all data, error bars show standard deviation of 50 resamplings with 50% of the data. Lines are given as guide to the eye. For full experimental statistics see Supplementary Table 5. Source data are provided as a Source Data file.

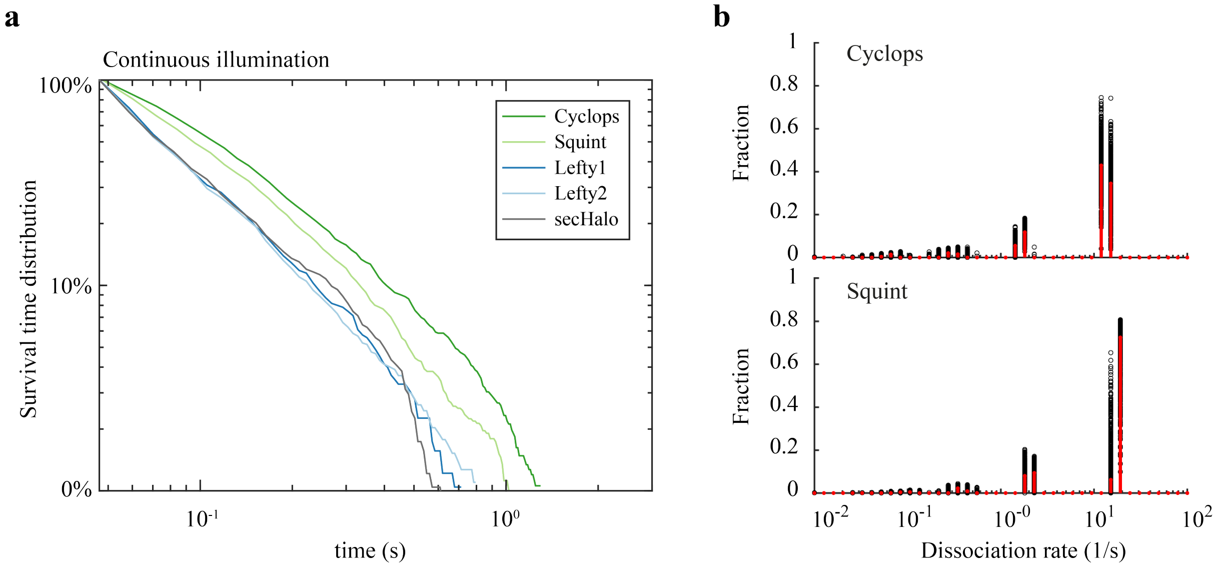

Supplementary Figure 6. Residence times in the extracellular space. a) Survival-time distributions of bound molecules obtained from continuous movies. **b)** Rate spectra of dissociation rates of Cyclops and Squint obtained by GRID using all data (red bars) and 500 resampling runs with randomly selected 80% of data (black data points) as an error estimation of the spectra. For full experimental statistics see Supplementary Table 6. Source data are provided as a Source Data file.

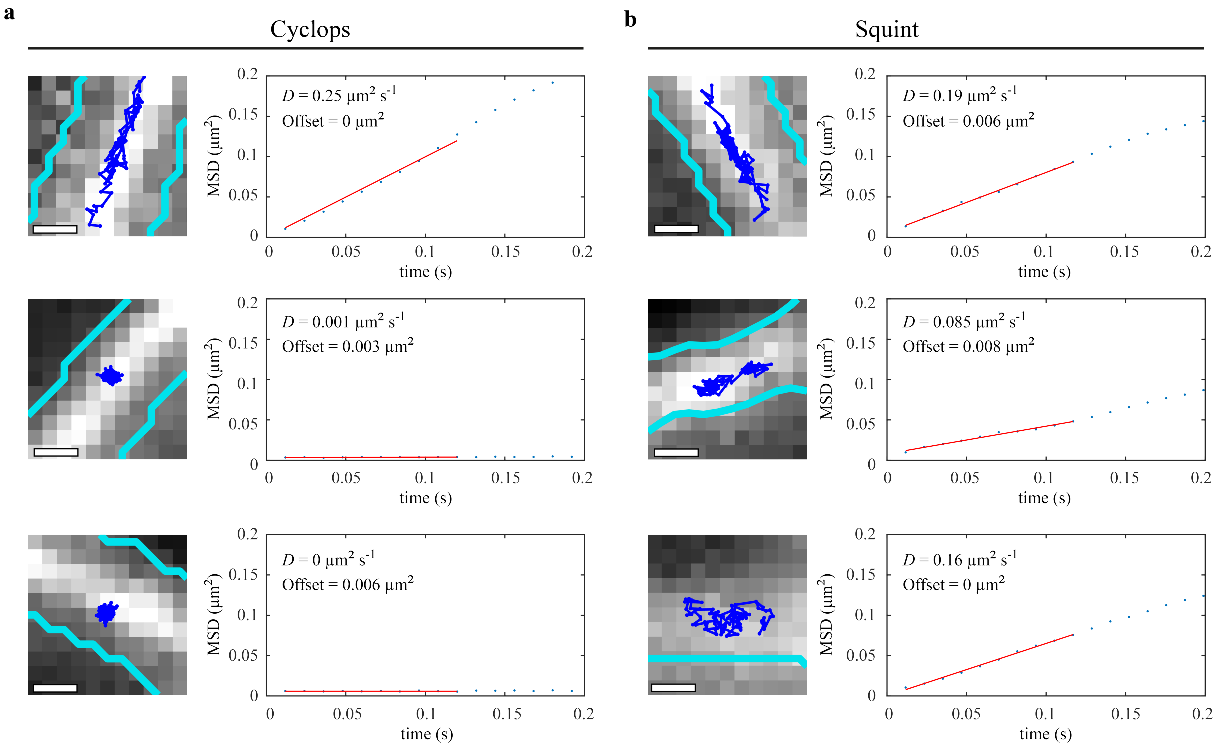

Supplementary Figure 7. Mean-squared displacement (MSD) analysis of an example set of extracellular binding events of Cyclops and Squint (Supplementary Movie 6 and 7). a,b) Left: memGFP signal averaged over 10 frames with overlaid track of a binding event (blue) with a minimum duration of 20 frames (234 ms) in the extracellular region (cyan). Right: MSD as a function of time (blue dots) with overlaid linear fit (red) for the determination of the diffusion coefficient of the tracks depicted on the left. Scale bars: 0.5 µm.

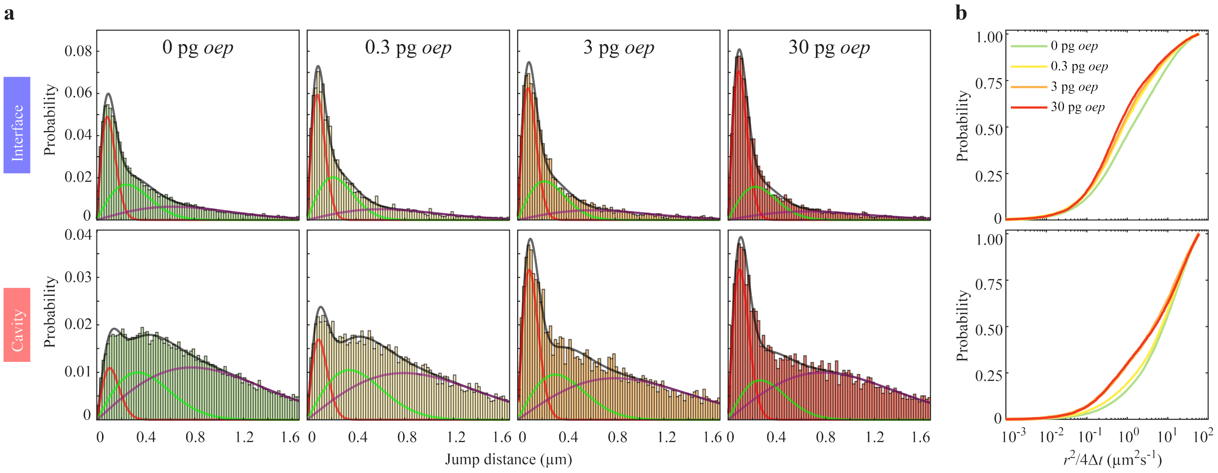

Supplementary Figure 8. Mobility of Squint decreases with overexpression of *oep*. a) Distribution of jump distances within single-molecule tracks in interfaces and cavities of Squint with the indicated amount of co-injected *oep*. Lines represent a three-component diffusion model (black) and the individual components (red, green, purple). **b)** Cumulative distributions of jump distances. For full experimental statistics see Supplementary Table 5. Source data are provided as a Source Data.

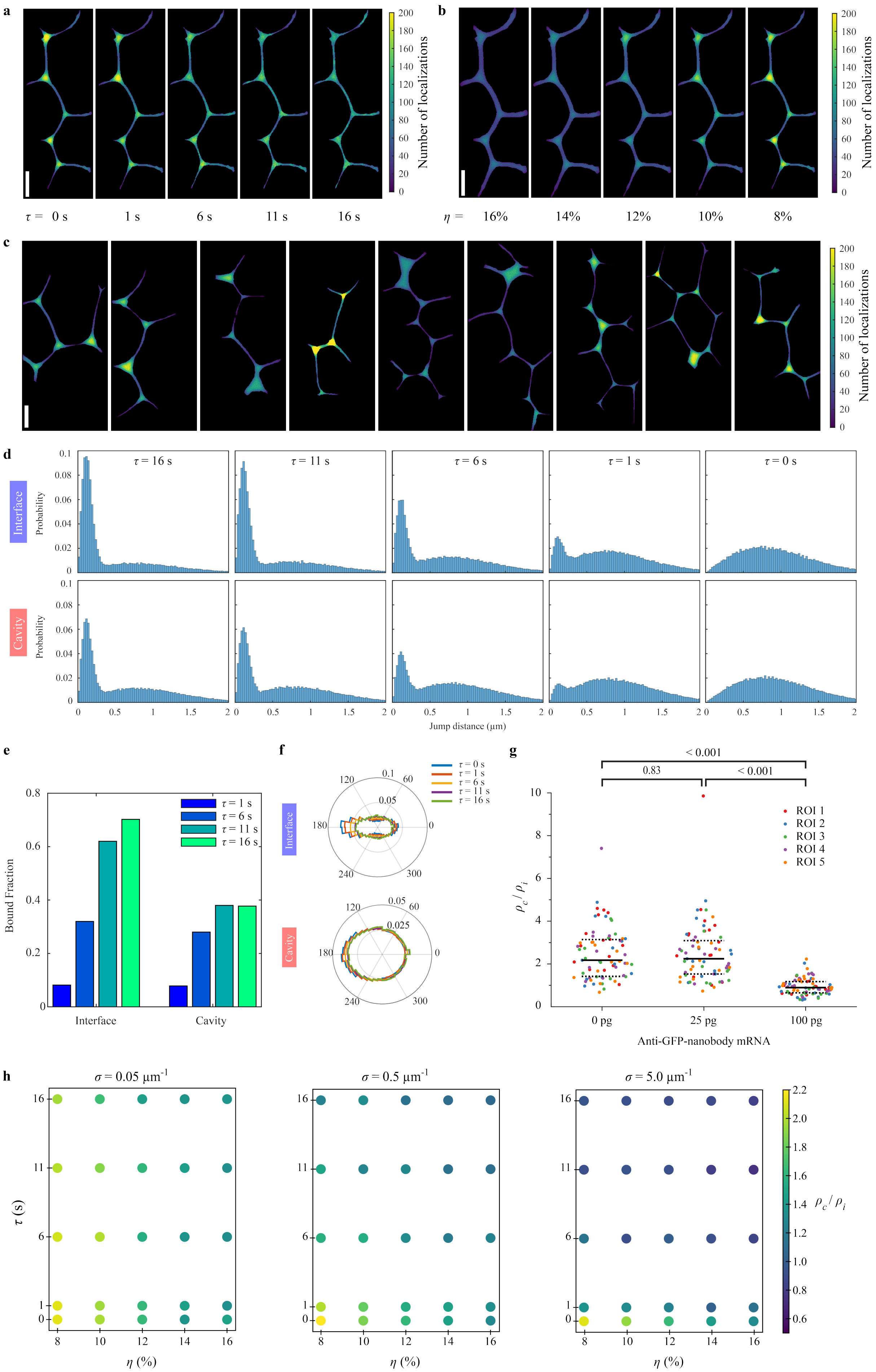
a
Number of localizations
τ = 0 s
1 s
6 s
11 s
16 s
b
η = 16%
14%
12%
10%
8%
c
d
Interface
Cavity
Probability
τ = 16 s
τ = 11 s
τ = 6 s
τ = 1 s
τ = 0 s
Jump distance (μm)
e
Bound Fraction
τ = 1 s
τ = 6 s
τ = 11 s
τ = 16 s
Interface
Cavity
f
τ = 0 s
τ = 1 s
τ = 6 s
τ = 11 s
τ = 16 s
g
< 0.001
0.83
< 0.001
ROI 1
ROI 2
ROI 3
ROI 4
ROI 5
ρc / ρi
0 pg
25 pg
100 pg
Anti-GFP-nanobody mRNA
h
σ = 0.05 μm-1
σ = 0.5 μm-1
σ = 5.0 μm-1
τ (s)
η (%)
ρc / ρi

Supplementary Figure 9. An agent-based model reveals key parameters affecting morphogen behavior in the extracellular space. a) Localization density plots of simulated morphogens with increasing residence times (τ) at σ =0.5 μm^{-1} and η = 8%. **b)** Localization density plots of simulated morphogens with decreasing extracellular fraction (η) at τ = 0 s, and σ = 0.5 μm^{-1}. **c)** Localization density plots of morphogens simulated on different geometries of the extracellular space; η = 8%, τ = 0 s, and σ = 0.5 μm^{-1}. **d)** Distribution of jump distances of simulated morphogens in interfaces and cavities for different residence times. **e)** Fraction of bound molecules in interfaces and cavities for different residence times. **f)** Distributions of angles between consecutive track segments of simulated morphogens in interfaces and cavities for different residence times. **g)** Ratio of mean GFP intensities in cavity and interface (ρ_c/ρ_i) for zebrafish embryos injected with different amounts of mRNA encoding membrane-tethered GFP-binding nanobody. Solid black lines indicate the median values, dashed black lines the 0.25 and 0.75 quantiles. The scattered dots show individual data points and are color-coded based on the regions of interest (ROIs) used for measurements. For each condition, fifteen cavity and interface regions each were measured from five ROIs. p-values (0 pg - 25 pg: 0.83; 0 pg - 100 pg: < 0.001; 25 pg - 100 pg: < 0.001) were calculated using the Kruskal-Wallis-Test. **h)** Two-dimensional representations of the three-dimensional scatter plot displayed in Figure 6b, showing the localization density ratios (ρ_c/ρ_i) for each of the simulated receptor densities σ (0.05 μm^{-1}, 0.5 μm^{-1}, and 5.0 μm^{-1}). Five residence times τ (0 s, 1 s, 6 s, 11 s, and 16 s) and five extracellular fractions η (8%, 10%, 12%, 14%, and 16%) were simulated for each receptor density condition. Scale bars in a, b and c are 10 μm. Source data are provided as a Source Data file for Fig. S9d,e,f,g.

Supplementary Tables

**Interface**	Cyclops	Squint	Lefty1	Lefty2	sec-Halo	sec-Halo-GFP
D_1 ($\mu m^2 s^{-1}$)	0.2 ± 0.02	0.32 ± 0.01	0.35 ± 0.04	0.43 ± 0.03	0.46 ± 0.04	0.49 ± 0.03
D_2 ($\mu m^2 s^{-1}$)	1.2 ± 0.1	2.6 ± 0.2	2.6 ± 0.3	3 ± 0.2	3.8 ± 0.3	3.6 ± 0.2
D_3 ($\mu m^2 s^{-1}$)	17 ± 1	17 ± 1	16 ± 1	17 ± 1	23 ± 1	19 ± 1
A_1, immobile	0.44 ± 0.03	0.35 ± 0.01	0.21 ± 0.02	0.24 ± 0.01	0.18 ± 0.01	0.21 ± 0.01
A_2, intermediate	0.39 ± 0.03	0.34 ± 0.01	0.39 ± 0.01	0.38 ± 0.01	0.38 ± 0.01	0.38 ± 0.01
A_3, fast	0.17 ± 0.01	0.31 ± 0.01	0.4 ± 0.02	0.37 ± 0.02	0.44 ± 0.02	0.4 ± 0.02

Supplementary Table 1. Diffusion parameters in interface regions obtained from fitting the cumulative distribution of jump distances of Cyclops, Squint, Lefty1 and Lefty2 shown in Figure 3.

**Cavity**	Cyclops	Squint	Lefty1	Lefty2	sec-Halo	sec-Halo-GFP
D_1 ($\mu m^2 s^{-1}$)	0.31 ± 0.02	0.45 ± 0.04	0.63 ± 0.11	0.73 ± 0.09	1.19 ± 0.15	0.6 ± 0.12
D_2 ($\mu m^2 s^{-1}$)	5.2 ± 0.7	4.8 ± 0.4	6.7 ± 0.7	5.5 ± 0.4	8.1 ± 0.9	5.5 ± 0.4
D_3 ($\mu m^2 s^{-1}$)	28 ± 3	26 ± 1	30 ± 2	27 ± 1	38 ± 3	28 ± 1
A_1, immobile	0.22 ± 0.01	0.09 ± 0.01	0.05 ± 0.01	0.06 ± 0.01	0.07 ± 0.01	0.03 ± 0.01
A_2, intermediate	0.3 ± 0.04	0.28 ± 0.02	0.31 ± 0.03	0.33 ± 0.02	0.34 ± 0.03	0.3 ± 0.02
A_3, fast	0.48 ± 0.04	0.63 ± 0.02	0.64 ± 0.04	0.61 ± 0.02	0.58 ± 0.04	0.66 ± 0.02

Supplementary Table 2. Diffusion parameters in cavity regions obtained from fitting the cumulative distribution of jump distances of Cyclops, Squint, Lefty1 and Lefty2 shown in Figure 3.

**Interface**	*oep* 0.3 pg	*oep* 3 pg	*oep* 30 pg
D_1 ($\mu m^2 s^{-1}$)	0.28 ± 0.02	0.31 ± 0.02	0.32 ± 0.02
D_2 ($\mu m^2 s^{-1}$)	1.9 ± 0.2	2 ± 0.3	2.2 ± 0.3
D_3 ($\mu m^2 s^{-1}$)	16 ± 1	16 ± 1	18 ± 1
A_1, immobile	0.4 ± 0.02	0.44 ± 0.03	0.5 ± 0.02
A_2, intermediate	0.35 ± 0.02	0.33 ± 0.02	0.29 ± 0.02
A_3, fast	0.25 ± 0.01	0.23 ± 0.02	0.21 ± 0.01

Supplementary Table 3. Diffusion parameters in interface regions obtained from fitting the cumulative distribution of jump distances of Squint upon overexpression of *oep* with 0.3 pg, 3 pg and 30 pg mRNA shown in Figure 5.

**Cavity**	*oep* 0.3 pg	*oep* 3 pg	*oep* 30 pg
D_1 ($\mu m^2 s^{-1}$)	0.37 ± 0.03	0.38 ± 0.03	0.38 ± 0.04
D_2 ($\mu m^2 s^{-1}$)	5.1 ± 0.6	4.1 ± 0.9	3 ± 1.1
D_3 ($\mu m^2 s^{-1}$)	27 ± 2	27 ± 4	26 ± 3
A_1, immobile	0.13 ± 0.01	0.25 ± 0.02	0.25 ± 0.03
A_2, intermediate	0.3 ± 0.03	0.24 ± 0.03	0.18 ± 0.02
A_3, fast	0.57 ± 0.03	0.51 ± 0.04	0.57 ± 0.03

Supplementary Table 4. Diffusion parameters in cavity regions obtained from fitting the cumulative distribution of jump distances of Squint with overexpression of *oep* with 0.3 pg, 3 pg and 30 pg mRNA shown in Figure 5

	Days	Embryos	Movies	Tracks			Jumps			
				Interface	Cavity	Combined	Interface	Cavity	Combined	Jumps/movie
Cyclops	7	32	128	2837	4063	6900	8569	8685	17254	135.5
Squint (S)	7	33	231	6903	15842	22745	31615	17983	49598	214.7
Lefty1	4	23	289	5012	18474	23486	32295	11050	43345	150.1
Lefty2	4	19	253	9365	28642	38007	62118	21539	83657	330.6
S + 0.3 pg *oep*	2	14	200	2987	6425	9412	12495	7070	19565	103.6
S + 3 pg *oep*	3	20	288	2875	3589	6464	6917	7721	14638	50.8
S + 30 pg *oep*	3	23	255	2729	3067	5796	5533	8224	13757	49.4
sec-Halo	3	20	281	9969	28655	38624	20585	52351	72936	259.6
sec-Halo-GFP	2	14	184	11385	29961	41346	25945	64001	89946	488.8

Supplementary Table 5. Statistics for continuous movie analysis.

	Movies	Tracks	Tracks per movie
Cyclops 12 ms	128	1418	11.1
Squint 12 ms	252	1782	7.1
Cyclops 58 ms	78	883	11.3
Squint 58ms	131	886	6.8
Cyclops 200 ms	91	986	10.8
Squint 200 ms	135	770	5.7
Cyclops 1000 ms	93	550	5.9
Squint 1000 ms	119	508	4.2

Supplementary Table 6. Statistics of single-molecule tracks.
n = 32 embryos in 7 days (Cyclops) and n = 42 embryos in 9 days (Squint) were measured.

	Number of movies in developmental stage						
	128-cell	256-cell	512-cell	1k	high	oblong	sphere
Cyclops	8	37	16	19	11	14	23
Squint	12	44	22	44	51	36	22
Lefty1	7	41	35	69	60	53	24
Lefty2	10	37	51	48	48	49	10

Supplementary Table 7. Statistics of movie numbers for different developmental stages.
