# Supplementary material for: Single-molecule tracking of Nodal and Lefty in live zebrafish embryos supports hindered diffusion model
Source: Nat Commun. 2022 Oct 15;13:6101. doi: 10.1038/s41467-022-33704-z (PMC9569377; doi:10.1038/s41467-022-33704-z)
Supplement: Supplementary file 3 — Description of Additional Supplementary Files [file 41467_2022_33704_MOESM3_ESM.pdf]

Description of Additional Supplementary Files

File Name: Supplementary Movie 1
Description: Visualization of the data shown in Figure 1. Left: single molecule imaging of Lefty2-Halo molecules imaged with 11.7 ms per frame. Center: memGFP signal averaged over 10 frames with overlaid interface (blue) and cavity (red) regions and tracked single molecules Right: TALM image showing the amount of detections in each pixel over the movie time. Scale bar: 10 μm.

File Name: Supplementary Movie 2
Description: Visualization of the data shown in Figure 2. Left: single molecule imaging of Halo-Cyclops at 11.7 ms per frame. Right: TALM image showing the amount of detections in each pixel over the movie time with overlaid interface (blue) and cavity (red) regions and tracked single molecules. Scale bar: 5 μm.

File Name: Supplementary Movie 3
Description: Visualization of the data shown in Figure 2. Left: single molecule imaging of Halo-Squint at 11.7 ms per frame. Right: TALM image showing the amount of detections in each pixel over the movie time with overlaid interface (blue) and cavity (red) regions and tracked single molecules. Scale bar: 5 μm.

File Name: Supplementary Movie 4
Description: Visualization of the data shown in Figure 2. Left: single molecule imaging of Lefty1-Halo at 11.7 ms per frame. Right: TALM image showing the amount of detections in each pixel over the movie time with overlaid interface (blue) and cavity (red) regions and tracked single molecules. Scale bar: 5 μm.

File Name: Supplementary Movie 5
Description: Visualization of the data shown in Figure 2. Left: single molecule imaging of Lefty2-Halo at 11.7 ms per frame. Right: TALM image showing the amount of detections in each pixel over the movie time with overlaid interface (blue) and cavity (red) regions and tracked single molecules. Scale bar: 5 μm.

File Name: Supplementary Movie 6
Description: Visualization of the data shown in Supplementary Figure 5. Top: single molecule imaging of Halo-Cyclops at 11.7 ms per frame. Bottom: memGFP signal averaged over 10 frames with overlaid extracellular region (cyan) and track of a binding event. Scale bar: 0.5 μm.

File Name: Supplementary Movie 7
Description: Visualization of the data shown in Supplementary Figure 5. Top: single molecule imaging of Halo-Squint at 11.7 ms per frame. Bottom: memGFP signal averaged over 10 frames with overlaid extracellular region (cyan) and track of a binding event. Scale bar: 0.5 μm.
