# Supplementary material for: Single-molecule tracking of Nodal and Lefty in live zebrafish embryos supports hindered diffusion model
Source: Nat Commun. 2022 Oct 15;13:6101. doi: 10.1038/s41467-022-33704-z (PMC9569377; doi:10.1038/s41467-022-33704-z)
Supplement: Supplementary file 11 — Reporting Summary [file 41467_2022_33704_MOESM11_ESM.pdf]

nature portfolio

Corresponding author(s): J. Christof M. Gebhardt
Patrick Müller

Last updated by author(s): Sep 23, 2022

Reporting Summary

Nature Portfolio wishes to improve the reproducibility of the work that we publish. This form provides structure for consistency and transparency in reporting. For further information on Nature Portfolio policies, see our Editorial Policies and the Editorial Policy Checklist.

Statistics

For all statistical analyses, confirm that the following items are present in the figure legend, table legend, main text, or Methods section.

n/a	Confirmed	
☐	☒	The exact sample size (*n*) for each experimental group/condition, given as a discrete number and unit of measurement
☐	☒	A statement on whether measurements were taken from distinct samples or whether the same sample was measured repeatedly
☐	☒	The statistical test(s) used AND whether they are one- or two-sided<br>*Only common tests should be described solely by name; describe more complex techniques in the Methods section.*
☒	☐	A description of all covariates tested
☒	☐	A description of any assumptions or corrections, such as tests of normality and adjustment for multiple comparisons
☐	☒	A full description of the statistical parameters including central tendency (e.g. means) or other basic estimates (e.g. regression coefficient) AND variation (e.g. standard deviation) or associated estimates of uncertainty (e.g. confidence intervals)
☐	☒	For null hypothesis testing, the test statistic (e.g. *F*, *t*, *r*) with confidence intervals, effect sizes, degrees of freedom and *P* value noted<br>*Give P values as exact values whenever suitable.*
☒	☐	For Bayesian analysis, information on the choice of priors and Markov chain Monte Carlo settings
☒	☐	For hierarchical and complex designs, identification of the appropriate level for tests and full reporting of outcomes
☒	☐	Estimates of effect sizes (e.g. Cohen's *d*, Pearson's *r*), indicating how they were calculated

Our web collection on statistics for biologists contains articles on many of the points above.

Software and code

Policy information about availability of computer code

Data collection	The diameter of the injection mix droplet was analyzed using the CellSens Entry 2.3 (Build 18987) imaging software.<br>Single molecule microscopy movies were acquired using Nikon NIS-Elements Version 4.40.00 64 bit.
Data analysis	Segmentation of extracellular regions in images was performed with ZeroCostDL4Mic (von Chamier et al., Nat Commun 12, 2276 (2021)).<br>Single-molecule tracking and analysis was performed in Matlab R2021a with the TrackIt Software (Kuhn et al., Sci Rep 11, 9465 (2021), https://gitlab.com/GebhardtLab/TrackIt, https://doi.org/10.5281/zenodo.7092296). Morphotrap experiments were analyzed in Fiji 2.9.0.<br>Statistical significance testing was performed in GraphPad Prism 9.0.1.<br>Agent-based modeling was performed in Python 3.7.12 (https://github.com/mueller-lab/morphogenDiffusion-ABM, https://doi.org/10.5281/zenodo.7104354).

For manuscripts utilizing custom algorithms or software that are central to the research but not yet described in published literature, software must be made available to editors and reviewers. We strongly encourage code deposition in a community repository (e.g. GitHub). See the Nature Portfolio guidelines for submitting code & software for further information.

Data

Policy information about availability of data

All manuscripts must include a data availability statement. This statement should provide the following information, where applicable:

- Accession codes, unique identifiers, or web links for publicly available datasets
- A description of any restrictions on data availability
- For clinical datasets or third party data, please ensure that the statement adheres to our policy

All single-particle tracking data and simulated tracks are freely available at https://doi.org/10.5061/dryad.9kd51c5kg. Source data for figures are provided with this paper in 'Source Data.xlsx'. Data supporting the findings of this manuscript will also be available from the corresponding authors upon reasonable request.

Human research participants

Policy information about studies involving human research participants and Sex and Gender in Research.

Reporting on sex and gender	N/A
Population characteristics	N/A
Recruitment	N/A
Ethics oversight	N/A

Note that full information on the approval of the study protocol must also be provided in the manuscript.

Field-specific reporting

Please select the one below that is the best fit for your research. If you are not sure, read the appropriate sections before making your selection.

☒ Life sciences ☐ Behavioural & social sciences ☐ Ecological, evolutionary & environmental sciences

For a reference copy of the document with all sections, see nature.com/documents/nr-reporting-summary-flat.pdf

Life sciences study design

All studies must disclose on these points even when the disclosure is negative.

Sample size	For each measurement condition of single-molecule data, >10 embryos were measured on several measurement days, resulting in hundreds of movies and thousand of tracks in each condition (see Supplementary Tables 5,6,7), sufficient to allow for a proper statistical comparison of different conditions. In our previous work (Reisser et al., Nature Communications 2018, https://doi.org/10.1038/s41467-018-07731-8), we found that a minimum of four different embryos yielded robust results.
Data exclusions	Embryos exhibiting drift due to cellular or whole embryo movements were excluded from analysis. Single-molecule tracks in regions, where the extracellular space was not clearly identifiable, were not taken into account.
Replication	Measurements were performed independently on a minimum of 2 measurement days. Replica measurements were performed successfully.
Randomization	For each measurement condition of single-molecule data, random embryos were selected. mRNA was injected independently into each embryo, the injected solution was the same for a measurement day. Replicas were measured at different days.<br>Therefore there is no randomization required to exclude any bias.
Blinding	Blinding was not required in this study. All data were recorded and analyzed under the same experimental conditions to exclude any bias.

Reporting for specific materials, systems and methods

We require information from authors about some types of materials, experimental systems and methods used in many studies. Here, indicate whether each material, system or method listed is relevant to your study. If you are not sure if a list item applies to your research, read the appropriate section before selecting a response.

Materials & experimental systems

n/a	Involved in the study
☒	☐ Antibodies
☒	☐ Eukaryotic cell lines
☒	☐ Palaeontology and archaeology
☐	☒ Animals and other organisms
☒	☐ Clinical data
☒	☐ Dual use research of concern

Methods

n/a	Involved in the study
☒	☐ ChIP-seq
☒	☐ Flow cytometry
☒	☐ MRI-based neuroimaging

Animals and other research organisms

Policy information about studies involving animals; ARRIVE guidelines recommended for reporting animal research, and Sex and Gender in Research

Laboratory animals	Danio rerio, Wild Indian Karyotype (WIK), Tübingen (TUE) and TE, aged 0.5-2 years.
Wild animals	The study did not involve wild animals.
Reporting on sex	Sex cannot be determined in zebrafish embryos, and sex-based information was not collected.
Field-collected samples	The study did not envolve samples collected from the fields.
Ethics oversight	Approved by the Regierungspräsidium Tübingen and the Regierungspräsidium Freiburg.

Note that full information on the approval of the study protocol must also be provided in the manuscript.
